# Supplementary material for: Modulation of protective reflex cough by acute immune driven inflammation of lower airways in anesthetized rabbits
Source: PLoS One. 2019 Dec 30;14(12):e0226442. doi: 10.1371/journal.pone.0226442 (PMC6936810; doi:10.1371/journal.pone.0226442)
Supplement: S1 Fig — Sensitization was induced by two intraperitoneal injections of OVA (200 μg/mL) in 2 weeks intervals. Dermal test was performed by two symmetrical intradermic injections of OVA (20 μg in 100 μL of saline) and of saline (NaCl 0.9%) one week after the last injection. Induration sizes (A) and erythema (B) were evaluated 24h and 48h after intradermic injections. (DOCX) [file pone.0226442.s001.docx]

**S1 Figure: Sensitization test to ovalbumin.**

Sensitization was induced by two intraperitoneal injections of OVA (200 µg/mL) in 2 weeks intervals. Dermal test was performed by two symmetrical intradermic injections of OVA (20 µg in 100 µL of saline) and of saline (NaCl 0.9%) one week after the last injection. Induration sizes (A) and erythema (B) were evaluated 24h and 48h after intradermic injections.

**A**

**NaCl**

**0.9%**

**OVA**

**200µg/mL**

0

500

1 000

1 500

2 000

2 500

**NaCl**

**0.9%**

**OVA**

**200µg/mL**

**24h**

**48h**

**Induration size (mm^2^)**

**B**

|  | **24h** | | **48 h** | |
| --- | --- | --- | --- | --- |
| **Erythema** | **NaCl**  **0.9%** | **OVA 200µg/mL** | **NaCl**  **0.9%** | **OVA 200µg/mL** |
| none | 16 | 9 | 12 | 7 |
| observed | 0 | 7 | 0 | 5 |
