# Supplementary material for: Modulation of protective reflex cough by acute immune driven inflammation of lower airways in anesthetized rabbits
Source: PLoS One. 2019 Dec 30;14(12):e0226442. doi: 10.1371/journal.pone.0226442 (PMC6936810; doi:10.1371/journal.pone.0226442)
Supplement: S1 Table — Preliminary results before study. Results are expressed as the mean ± SD of number of counts indicated in brackets. The number of rabbits of the different groups is indicated in column entitled n. (DOCX) [file pone.0226442.s002.docx]

**S1 Table: Percentage of macrophages and eosinophils in BAL of rabbits following different OVA exposure procedures. Preliminary results before study.**

Results are expressed as the mean ± SD of number of counts indicated in brackets. The number of rabbits of the different groups is indicated in column entitled n.

|  |  | macrophages | eosinophils |
| --- | --- | --- | --- |
|  | n | % of cell count in BAL | |
| Saline sensitized and 1 day saline aerosol | 1 | 97.7 ± 1.6 (4) | 2.3 ± 1.6 (4) |
| OVA sensitized + 1 day saline aerosol | 2 | 98.6 ± 1.2 (8) | 1.3 ± 1.2 (8) |
| OVA sensitized + 2 days saline aerosol | 2 | 94.3 ± 6.3 (8) | 5.5 ± 6 (8) |
| OVA sensitized + 1 day OVA aerosol | 1 | 91.6 (2) | 8.4 (2) |
| **OVA sensitized + 2 days OVA aerosol** | 1 | 61.2 (2) | **29.4 (2)** |
